# Genomic Characterization and Pathogenicity of BJEU06-1-Like PRRSV-1 ZD-1 Isolated in China

**DOI:** 10.1155/2023/6793604

**Published:** 2023-03-27

**Authors:** Hu Xu, Bangjun Gong, Qi Sun, Chao Li, Jing Zhao, Lirun Xiang, Wansheng Li, Zhenyang Guo, Yan-dong Tang, Chaoliang Leng, Zhen Li, Qian Wang, Guohui Zhou, Tongqing An, Xuehui Cai, Zhi-Jun Tian, Jinmei Peng, Hongliang Zhang

**Affiliations:** ^1^State Key Laboratory of Veterinary Biotechnology, Harbin Veterinary Research Institute, Chinese Academy of Agricultural Sciences, Harbin 150001, China; ^2^Henan Key Laboratory of Insect Biology in Funiu Mountain, Henan Provincial Engineering Laboratory of Insects Bio-reactor, China-UK-NYNU-RRes Joint Laboratory of Insect Biology, Nanyang Normal University, Nanyang 473061, China; ^3^Pingdingshan Center for Animal Disease Control and Prevention, Pingdingshan 467000, China

## Abstract

Porcine reproductive and respiratory syndrome virus (PRRSV)-1 and PRRSV-2 have long been cocirculating in China. To date, all PRRSV-1 strains in China have been classified as subtype 1. We investigated the prevalence of PRRSV-1 in several areas of China from 2016 to 2022 and found that BJEU06-1-like strains comprised the main epidemic branch of PRRSV-1. Pathogenicity data for this subgroup are currently lacking. In this study, the Chinese BJEU06-1-like PRRSV-1 strain ZD-1 was isolated from primary alveolar macrophages (PAMs). ZD-1 has undergone no recombination and has a 5-aa discontinuous deletion in the Nsp2 protein, similar to other BJEU06-1-like strains; additionally, ZD-1 has a 26 aa C-terminal truncation in the GP3 gene. Pathogenicity studies revealed that ZD-1 causes obvious clinical symptoms: prolonged fever; reduced body weight; alveolar epithelial proliferation and moderate alveolar diaphragm widening in the lungs; diffuse lymphocytic hyperplasia in the lymph nodes; high levels of viremia in the serum; and elevated viral loads in the lungs, lymph nodes, and tonsils. These results suggested that the BJEU06-1-like PRRSV-1 strain ZD-1 is moderately pathogenic to piglets. This is the first study to evaluate the pathogenicity of the BJEU06-1-like branch in China, enriching the understanding of PRRSV-1 in China.

## 1. Introduction

Porcine reproductive and respiratory syndrome (PRRS) is caused by PRRS virus (PRRSV), an enveloped, positive-sense, single-stranded RNA virus belonging to the genus *Betaarterivirus*, family *Arteriviridae*, and order *Nidovirales* [1]. PRRS affects swine production and breeding in major swine-producing countries and is associated with tremendous economic losses [2]. PRRSV is divided into two species: *Betaarterivirus suid* 1 (PRRSV-1) and *Betaarterivirus suid* 2 (PRRSV-2) [1]. Lelystad virus (LV) and VR-2332 are representative strains of PRRSV-1 and PRRSV-2, respectively, which share 60% nucleotide identity at the whole-genome level [3, 4]. Although PRRSV-2 is predominant in China, PRRSV-1 isolates have also existed there for more than twenty years [5–11].

Based on differences in the ORF5 and ORF7 genes, PRRSV-1 strains can be subdivided into 4 subtypes, subtype 1 to subtype 4 [12–15]. Interestingly, only subtype 1 has spread to continents other than Europe [15]. The remaining subtypes have been reported only in Eastern European countries and Russia [15]. Similarly, all the isolated strains in China belong to subtype 1 and can be further divided into four subgroups [6]: BJEU06-1-like, Amervac-like, HKEU16-like, and NMEU09-1-like. To the best of our knowledge, only GZ11-G1 and HLJB1, both of which belong to the Amervac-like subgroup, have been evaluated for pathogenicity [16, 17]. The pathogenicity of BJEU06-1-like subgroup strains has not been evaluated to date.

In the present study, we carried out molecular epidemiological surveillance of PRRSV-1 strains from 2016 to 2022. In addition, we isolated one BJEU06-1-like PRRSV-1 strain, ZD-1, from a pig farm, determined its complete genomic sequence, and analyzed the genomic genetic variation of the virus and its pathogenicity in piglets.

## 2. Materials and Methods

### 2.1. Sample Collection and Genome Sequencing

From 2016 to 2022, we collected more than 3200 clinical samples (including lung, lymph node, and serum samples) from pigs with suspected PRRSV infections from different pig farms in 16 provinces in China (Heilongjiang, Jilin, Liaoning, Shandong, Henan, Guangdong, Guangxi, Zhejiang, Hebei, Hubei, Xinjiang, Inner Mongolia, Tianjin, Sichuan, Jiangxi, and Jiangsu). The disease materials come mainly from pig farmers and small-scale pig farms; some of the pigs at the included farms showed clinical symptoms such as miscarriage or stillbirth (Table 1). Tissue sample processing, RNA extraction, cDNA preparation, and RT-PCR were performed as described in previous reports [18]. The primers used to detect the PRRSV-1 ORF5 gene have also been reported previously [19]. Eight pairs of primers amplifying overlapped fragments were selected from our previous study and used for ZD-1 complete genome sequencing [19].

The PCR products were purified using a gel extraction kit (Tiangen, China) and cloned into the PMD18-T cloning vector (TaKaRa, Japan). The cloned products were transformed into DH5*α* competent cells (Tiangen, China), and three positive clones for each fragment were selected for Sanger sequencing (Kumei, China). The obtained sequences were assembled using Lasergene software (DNASTAR Inc.).

### 2.2. Sequencing Analysis

The deduced amino acid sequences were aligned by ClustalW with Lasergene software (DNASTAR Inc.) [20]. The ORF5 sequences of the reference strains collected from the GenBank database and the PRRSV-1 strains obtained in this study were aligned using MAFFT [21]. The phylogenetic tree was inferred using the maximum likelihood method and the GTR model (MEGA 7.0) [22]. The topology of the trees was confirmed with 1000 bootstrap replication steps [23]. The generated phylogenetic tree was annotated using the online software ITOL (https://itol.embl.de/) [24]. To test the role of recombination in the generation of ZD-1, the multiple alignments of the genomes were submitted to Recombination Detection Program 4 (RDP4) to screen for potential recombination events [25]. Potential recombination events were tested by seven different algorithms (RDP, GeneConv, BootScan, MaxChi, Chimera, SiScan, and 3Seq) with Bonferroni correction. The detection of a recombination event by four or more of the seven methods implemented in RDP4 was considered significant evidence for recombination [9]. The recombination breakpoints were further analyzed by the Genetic Algorithm for Recombination Detection (GARD) and SimPlot software v.3.5.1 [26, 27].

### 2.3. Virus Isolation

PAMs were obtained from 5-week-old specific-pathogen-free (SPF) pigs and cultured in RPMI 1640 medium (Gibco BRL Co. Ltd., USA) supplemented with 10% fetal bovine serum (FBS; ExCell Bio., Australia) [28]. PRRSV-1-positive samples were homogenized in Dulbecco's modified Eagle's medium (DMEM, Gibco). Supernatants were collected after centrifugation and filtered through 0.45-*μ*m filters and then inoculated into porcine PAMs obtained from a PRRSV-negative pig as described [29]. Three days later, the cultures were harvested and stored at −80°C as viral stocks. Cultures of the third passage in PAMs were used for animal experiments. The viral titer was assessed as described in previous reports [30].

### 2.4. Immunofluorescence Assay (IFA)

IFAs were conducted as previously described [31]. Viral antigens were prepared by inoculating PAMs with PRRSV-2 HUN4 (GenBank accession: EF635006.1) and the BJEU06-1-like PRRSV isolate ZD-1. Monoclonal antibodies against PRRSV-1 EU-1 (Mab EU-1; stored in our laboratory) and against PRRSV-2 3F7 were added to the cells and incubated for 30 min [32]. The cells were washed three times with PBS and then incubated with goat antimouse IgG antibody conjugated with FITC (Sigma, USA) and secondary antibodies for 30 min. Finally, the cells were washed three times with PBS and observed under a fluorescence microscope while in PBS.

### 2.5. Animal Experiments

Eight 21-day-old PRRSV-free piglets obtained from PRRS-free farms in Harbin were randomly divided into 2 groups: a challenge group (*n* = 5) and a control group (*n* = 3). The piglets in the challenge group and control group were inoculated intramuscularly (2 mL) and intranasally (2 mL) with ZD-1 (1 × 10^4^ TCID_50_/mL, with 4 mL per pig) and RPMI 1640, respectively. Blood was collected at 0, 3, 7, 10, 14, 17, and 21 days postinfection (dpi) for viremia detection via real-time quantitative PCR (RT‒qPCR) using a method established by our laboratory that has not yet been published. PRRSV-specific antibodies were measured using a commercial ELISA kit (IDEXX, Inc., Westbrook, ME, USA) according to the manufacturer's instructions. Based on the manufacturer's guidelines, sample-to-positive control (S/P) ratios greater than 0.4 were considered positive.

Clinical signs and rectal temperatures were recorded daily. The body weights of piglets were measured at 0, 7, 14, and 21* *dpi. All of the piglets were euthanized at 21* *dpi. To determine the distribution of PRRSV-1 (ZD-1) in the infected piglets, heart, liver, spleen, lung, kidney, lymph node (mandibular), tonsil, small intestine, brain, and stomach tissue samples were obtained for viral detection by RT-qPCR. In addition, lung and lymph node (mandibular) samples of each piglet were collected at 21* *dpi, fixed in 4% formaldehyde solution, and further processed for histopathology using hematoxylin and eosin staining.

### 2.6. Statistical Analysis

Significant differences between two groups were determined using a *t*-test (and nonparametric tests) in GraphPad 5.0 (San Diego, CA, USA). The level of significance was set at *p* < 0.05 [33].

## 3. Results

### 3.1. PRRSV-1 Detection Results and Phylogenetic Analysis

**Figure 1:**
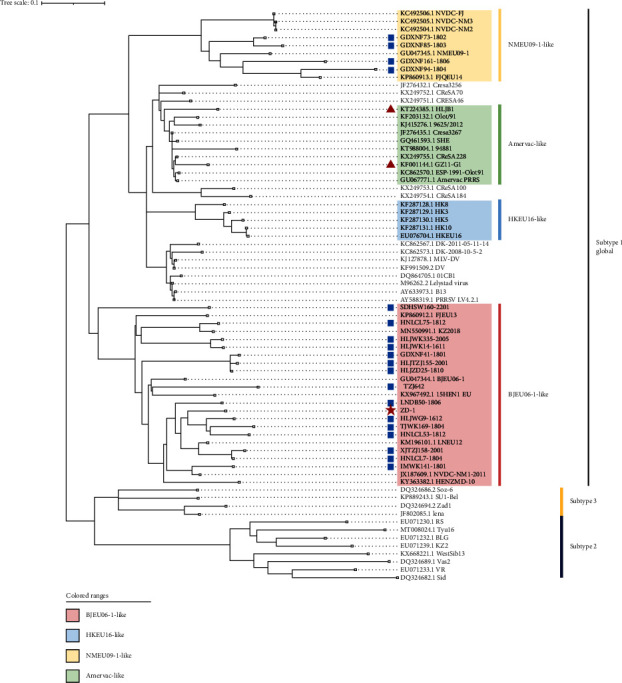
Phylogenetic analysis of the nucleotide sequences of ORF5 of PRRSV-1 strains. Chinese PRRSV-1 isolates belong to subtype 1 (global) and can be divided into four subgroups (Amervac-like, BJEU06-1-like, HKEU16-like, and NMEU09-1-like). Recently obtained Chinese PRRSV-1 sequences in our lab are represented by blue squares (■). The ZD-1 isolate from this study is indicated by a red star (★). Two Chinese PRRSV-1 isolates, HLJB1 and GZ11-G1, used in the pathogenicity evaluation are labeled with red triangles (▲).

A total of 20 ORF5 sequences of PRRSV-1 were obtained by RT-PCR from 2016 to 2022. All positive samples of PRRSV-1 strains were from the provinces of Heilongjiang, Guangdong, Tianjin, Xinjiang, Shandong, Henan, Liaoning, and Inner Mongolia (Table 1). To understand the genetic relationships of the newly identified PRRSV-1 strains and other representative strains, phylogenetic analysis was performed based on the nucleotide sequences of the ORF5 genes, as shown in Figure 1. According to the phylogenetic tree, all new Chinese PRRSV-1 strains are clustered with subtype 1 (Figure 1). As previously reported [34], Chinese PRRSV-1 strains are clustered mainly into four subgroups within subtype 1, namely, Amervac-like, BJEU06-1-like, HKEU16-like, and NMEU09-1-like strains. Four strains, GDXNF73-1802, GDXNF85-1803, GDXNF94-1804, and GDXNF161-1806, were classified as NMEU09-1-like, and the remaining 16 strains (ZD-1, HLJWK14-1611, HLJWG9-1612, IMWK141-1801, GDXNF41-1801, HNLCL7-1804, TJWK169-1804, LNDB50-1806, HLJZD25-1810, HNLCL53-1812, HNLCL75-1812, HLJTZJ155-2001, XJTZJ158-2001, HLJWK335-2005, TZJ642, and SDHSW160-2201) were classified as a BJEU06-1-like subgroup (Figure 1). Therefore, BJEU06-1-like strains are the main epidemic strains of PRRSV-1 in some areas of China.

### 3.2. Virus Isolation and Identification

**Figure 2:**
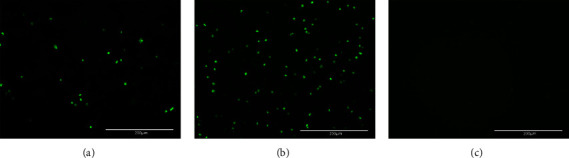
IFAs showing the reactivity of a monoclonal antibody against PRRSV-1 to ZD-1-P3-infected (a), HUN4-infected (b), and control (c) PAMs. Scale bar = 200 *μ*m.

To evaluate the pathogenicity of Chinese PRRSV-1 strains, tissue homogenates of samples positive for BJEU06-1-like strains were added to primary alveolar macrophages (PAMs). However, the immunofluorescence assay (IFA) results indicated that only ZD-1 could replicate in PAMs for 3 passages (Figure 2). To confirm this result, 19 other PRRSV-1-positive samples were blindly passaged in PAMs for over five passages. Notably, the other 19 PRRSV-1-positive samples in this research could not be isolated from PAMs.

### 3.3. Genomic Characteristics of ZD-1

**Figure 3:**
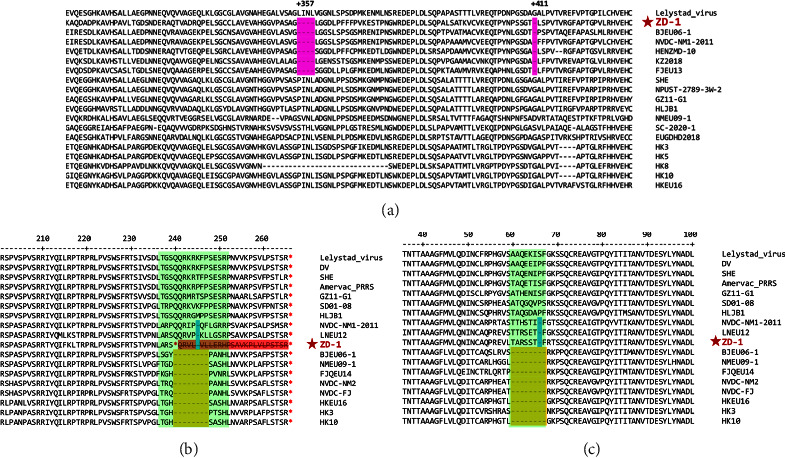
The alignment of Nsp2, GP3, and GP4 protein aa sequences of Chinese PRRSV-1 strains. (a) Alignment of the partial Nsp2 aa sequences of PRRSV-1 strains. Purple indicates the characteristic BJEU06-1-like PRRSV-1 5-aa (4 + 1) discontinuous deletion. (b) Alignment of the partial ORF3 aa sequences of Chinese PRRSV-1 strains. (c) Alignment of the partial ORF4 aa sequences of Chinese PRRSV-1 strains. The aa deletion regions are marked in blue or yellow. Two mutational hotspots, 237–352 in GP3 and 60–67 in GP4, are indicated by green boxes. Termination codons are indicated by an asterisk (^∗^). The 26 aa C-terminal truncation mutants are shaded in red. The ZD-1 strain is marked with a red star (★).

To further characterize ZD-1, we sequenced the whole genome of this strain. The complete genome of ZD-1 was 15,082 nucleotides (nt) in length, excluding the poly (A) tail. Homology analysis with representative PRRSVs revealed that ZD-1 shared the highest genomic sequence homology with BJEU06-1 (89.4%); ZD-1 exhibited 87.1%, 85.3%, 83.4%, 86.2%, 85.9%, 88.9%, and 60.8% identity with Amervac PRRS, HKEU16, NMEU09-1, HLJB1, GZ11-G1, LV, and VR-2332, respectively. Partial Nsp2 sequence alignment showed that ZD-1 has a 5-aa (4 + 1) discontinuous deletion corresponding to residues 357–360 and 411 of Lelystad Nsp2, which is consistent with the patterns observed in BJEU06-1-like strains (Figure 3(a)). It was previously reported that there were mutational hotspots at aa positions 237–252 of GP3 and aa positions 60–67 of GP4 in the overlapping regions of GP3 and GP4 [35]. Based on the amino acid alignment of GP3 and GP4, the ZD-1 strain had two 1-aa deletions: one was located at aa position 245 of GP3 (Figure 3(b)), and the other was located at aa position 66 of GP4 (Figure 3(c)). Notably, the ZD-1 strain had an early termination of 26 amino acids in GP3 (Figure 3(b)).

To determine whether recombination events played a role in the generation of ZD-1, possible recombination events were examined in SimPlot, RDP4, and GARD software. The results showed that there were no obvious recombination signals (data not shown).

### 3.4. Clinical Signs of ZD-1-Infected Piglets

**Figure 4:**
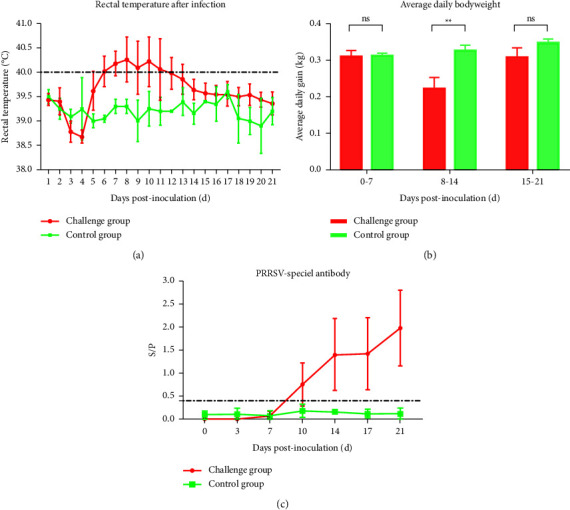
Rectal temperatures, average daily body weights, and PRRSV-specific antibody levels in the experimental piglets. (a) A rectal temperature ≥40.0°C was defined as fever. The mean ± SD (error bars) of temperatures is shown. (b) The body weight gain (kg) of piglets was calculated at 0, 7, 14, and 21 dpi. The mean ± SD (error bars) of body weight gain is shown. ^∗∗^, *P* < 0.01; ns, no significant difference. (c) Pig serum was analyzed for PRRSV-specific antibodies. The threshold for seroconversion was set at a sample-to-positive (S/P) ratio of 0.4. The bars represent the average S/P of one group of piglets. The mean ± SD (error bars) of the specific antibodies is shown.

Piglets infected with ZD-1 exhibited fever (rectal temperature exceeding 40.0°C) beginning at 5 dpi and persisting until 11 dpi, with a body temperature peak (40.9°C) at 10 dpi; thereafter, the rectal temperature recovered gradually (Figure 4(a)). The rectal temperature of uninfected piglets remained normal throughout the trial. Clinical observations revealed that ZD-1-infected piglets exhibited clinical signs such as inappetence, lethargy, tachypnea, and cough. By contrast, the piglets in the control group behaved normally without any clinical signs throughout the experiment. The body weight of the piglets was measured at 0, 7, 14, and 21 dpi, and the results showed that the average daily body weight gain in ZD-1-infected piglets was significantly lower (*p* < 0.01) than that in uninfected piglets at 8∼14 dpi (Figure 4(b)). All the piglets survived until the end of the experiment.

### 3.5. Antibody Detection Postinfection

Blood samples were collected at 0, 3, 7, 10, 14, 17, and 21 dpi to measure PRRSV-specific antibodies using a commercially available enzyme-linked immunosorbent assay (ELISA) (IDEXX Laboratories, U.S.A.) kit. PRRSV-specific antibodies were detected in four of five ZD-1-infected pigs at 10 dpi and in all infected pigs since 14 dpi (Figure 4(c)). In PRRSV-infected pigs, the S/P ratio gradually increased until the end of the experiment, whereas no PRRSV-specific antibodies were detected in the control animals (Figure 4(c)).

### 3.6. Macroscopic and Histopathological Lesions

**Figure 5:**
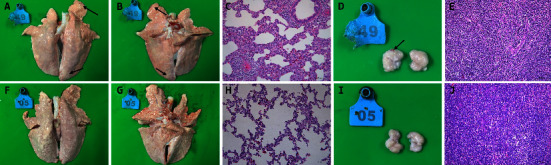
Pathological lesions of the lung and submaxillary lymph nodes. All piglets were euthanized at 21 dpi. Mild interstitial pneumonia with consolidation in the lungs in the challenge group (a, b) and hemorrhage in the lymph nodes (d) were observed when compared with the control group (f, g, i). Interstitial pneumonia characterized by extensive inflammatory cell infiltration with alveolar epithelial proliferation and moderate alveolar diaphragm widening in the lungs could be observed in challenge group pigs (c) when compared with control groups (h). Compared with the control group pigs (j), lymphocytic hyperplasia in the lymph nodes was observed in the challenge group pigs (e). Original magnification, 200x.

Necropsies were performed at 21 dpi. Compared with the piglets in the control group, those in the challenge group showed lesions typical of PRRS, such as consolidation in the lungs and hemorrhaging in the lymph nodes (3/5) (Figures 5(a), 5(b), and 5(d)). Furthermore, histopathology of those in the challenge group revealed extensive inflammatory cell infiltration with alveolar epithelial proliferation and moderate alveolar diaphragm widening in the lungs (5/5) (Figure 5(c)) and diffuse lymphocytic hyperplasia in the lymph nodes (Figure 5(e)). No pathological lesions were observed in the above-described tissues of pigs in the control group.

### 3.7. Viremia and Viral Loads in Tissue Assessment

**Figure 6:**
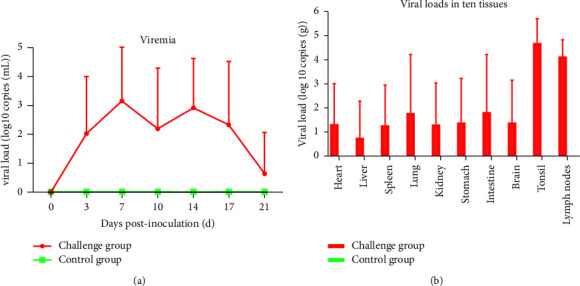
Viral load and distribution in tissues. The PRRSV viral load in tissues and serum from each group were determined using RT-qPCR. Tissue samples were collected at 21 dpi, while serum was collected at 0, 3, 7, 10, 14, 17, and 21 dpi. (a) Serum viral load on different collection dates. (b) Viral loads in different tissues of ZD-1-infected piglets. The data are presented as mean ± SD (error bars).

To further evaluate the difference in viremia and distribution in ten tissues among different groups, serum samples from 0, 3, 7, 10, 14, 17, and 21 dpi and ten organ tissues were evaluated using RT-qPCR. As illustrated in Figure 6(a), the viral load of the ZD-1 challenge group increased beginning at 3 dpi and peaked at 7 dpi. No viremia was detected in the serum samples from the control group throughout the study. Part of the ORF7 gene was sequenced to confirm that the samples contained the original virus (data not shown).

The hearts, livers, spleens, lungs, kidneys, lymph nodes (mandibular), tonsils, small intestines, brains, and stomachs were collected from ZD-1-infected piglets and analyzed by RT-qPCR. The viral load differed in different tissues (Figure 6(b)). The highest viral load was detected in the tonsil, followed by the lymph nodes (mandibular). In these two tissues, a higher viral load was detected in ZD-1-infected piglets, and the viral copy number detected was similar in all the infected piglets.

## 4. Discussion

PRRSV-1 and PRRSV-2 have coexisted in China for more than 25 years [11]. Prior to 2011, almost all PRRSV isolates from China reported in the literature belonged to PRRSV-2. However, since 2011, PRRSV-1 has not only become widely distributed in China but also now comprises four subgroups according to phylogenetic analysis, namely, the BJEU06-1-like, Amervac-like, HKEU16-like, and NMEU09-1-like subgroups [11, 34, 36–40]. In this study, we investigated the prevalence of PRRSV-1 in some areas of China from 2016 to 2022 and obtained a total of 20 PRRSV-1 ORF5 gene sequences. Phylogenetic analysis showed that all of the sequences belonged to the BJEU06-1-like and NMEU09-1-like subgroups; specifically, 16 strains, including ZD-1, belonged to the BJEU06-1-like subgroup, and the other 4 strains belonged to the NMEU09-1-like subgroup. According to our results, BJEU06-1-like strains are the main epidemic strains of PRRSV-1 in some areas of China. However, to the best of our knowledge, only GZ11-G1 and HLJB1 PRRSV-1 strains have been tested for pathogenicity in China, both of which are Amervac-like strains [16, 17]. Therefore, evaluating the pathogenicity of the BJEU06-1-like subgroup is of great significance for understanding the harmful effects of PRRSV-1 on the pig breeding industry in China. In this study, we successfully isolated one BJEU06-1-like strain (ZD-1), and the genome sequence and pathogenicity of ZD-1 were analyzed.

The Nsp2 gene has been shown to be highly variable in length among the different sequenced isolates of PRRSV and can tolerate a number of mutations, insertions, and deletions [41]. Compared with that of the Lelystad strain, the Nsp2 protein of ZD-1 has a discontinuous deletion of 5 aa (4 + 1), similar to other BJEU06-1-like strains. GP3 contains a hypervariable region located in the carboxyl-terminal end that overlaps with ORF4 [35]. Previous studies indicated that this region may be subject to more rapid changes than other regions during immune selective pressure [42]. Amino acid alignment of the highly variable region of GP3 and GP4 of ZD-1 with those of other PRRSV-1 isolates showed that this virus had one amino acid deletion at position 245 that was similar to those in LNEU12 and NVDC-NM1-2011 strains isolated in China. Importantly, ORF3 in the ZD-1 strain had a 26 aa truncation, resulting in a premature stop codon. The 18 aa and 21 aa truncations at the C-terminus were previously reported in PRRSV-1 in North America and Europe [43, 44]. To our knowledge, a PRRSV-1 strain with premature termination of amino acids of GP3 has not been reported in China. Additionally, our study is the first to report a 26 aa truncation at the C-terminus of GP3 in a PRRSV-1 strain. The highly pathogenic PRRSV-1 strain Lena also has a premature termination at the C-terminus of GP3 [45]. It remains unknown whether these truncations cause increased viral pathogenicity.

PRRSV-1 strains are genetically diverse and cause highly variable clinical symptoms. PRRSV-1 subtype 1 strains are circulating widely in Europe, Asia, and North America, and most of them have low pathogenicity [46–48]. However, recently, some countries have reported the emergence of highly pathogenic PRRSV-1 subtype 1 strains [49–51]. Strains of PRRSV-1 subtype 2 have been demonstrated to be more virulent than PRRSV-1 subtype 1 strains [52]. Subtype 3 of the PRRSV-1 strain “Lena-like,” which has antigenic heterogeneity with other subtype strains, has been shown to be highly pathogenic in pigs [45]. The Chinese Amervac-like strains GZ11-G1 and HLJB1 have been reported to have lower pathogenicity in infected piglets [16, 17]. To explore the pathogenicity of BJEU06-1-like PRRSV, five 21-day-old piglets were inoculated with the ZD-1 strain. Our results showed that the piglets exposed to ZD-1 exhibited typical clinical signs: the pigs started to show fever at 5 dpi, the rectal temperature exceeded 40.0°C, and a fever temperature remained until 13 dpi. Pigs had significantly longer fevers than those infected with the GZ11-G1 and HLJB1 strains (Table 2). The infected pigs had consolidation in the lung and hemorrhaging in the lymph nodes. Histopathology revealed extensive inflammatory cell infiltration with alveolar epithelial proliferation, moderate alveolar diaphragm widening in the lungs, and diffuse lymphocytic hyperplasia in the lymph nodes in the challenge group. A high level of viremia is one of the most typical findings for highly pathogenic strains [45, 53, 54]. The highest viral titer in serum samples of the ZD-1-infected animals was higher than that of the GZ11-G1 strain. In addition, the duration of viremia was longer than that of the HLJB1 strain. Therefore, ZD-1, a BJEU06-1-like strain, is moderately pathogenic and more virulent than GZ11-G1 and HLJB1, which are Amervac-like strains.

However, there have been many reports of highly pathogenic PRRSV-1 in Europe. The PRRSV-1 strains PR40/2014 (subtype 1), WestSib13 (subtype 2), and Lena (subtype 3) have been shown to be highly pathogenic. PRRSV-1 is generally regarded as a low-pathogenicity virus in China [45, 49, 52]. Although ZD-1 is more pathogenic than other Chinese PRRSV-1 strains, its pathogenicity is significantly lower than that of PRRSV-1, which has high pathogenicity in Europe. China, the world's largest importer of breeding pigs, has been threatened by the cross-border importation of PRRSV strains. Chinese customs intercepted pigs infected with PRRSV-1 as early as 1997 [55]. Therefore, it is of great significance for the protection of the pig industry in China to increase the detection of PRRSV-1.

In summary, BJEU06-1-like strains are the main circulating strains of PRRSV-1 in some parts of China. We isolated and identified the BJEU06-1-like PRRSV-1 ZD-1 strain. Genomic characterization showed that ZD-1 had the same 5-aa discontinuous deletion in Nsp2 as other BJEU06-1-like strains and a 26 aa premature truncation in the C-terminus of GP3. Its pathogenicity is moderate in piglets and is higher than that of other PRRSV-1 strains in China. Our findings contribute to an understanding of the evolutionary characteristics of Chinese PRRSV-1 strains and provide data on BJEU06-1-like isolates in China.

## Figures and Tables

**Table 1:** Information on Chinese PRRSV-1 isolates.

No.	Taxa	Accession no.	Isolated year	Clinical signs	Pig farm size	Province	Gene region
1	ZD-1	OP355712	2016	Sow abortion rate of 10% and piglet mortality rate of 10%	100	Heilongjiang	Whole genome
2	HLJWK14-1611	OP302768	2016	Unknown	Unknown	Heilongjiang	ORF5
3	HLJWG9-1612	OP302771	2016	Unknown	Unknown	Heilongjiang	ORF5
4	IMWK141-1801	OP302776	2018	Unknown	Unknown	Inner Mongolia	ORF5
5	GDXNF41-1801	OP302766	2018	Unknown	Unknown	Guangdong	ORF5
6	GDXNF73-1802	OP302762	2018	Unknown	Unknown	Guangdong	ORF5
7	GDXNF85-1803	OP302761	2018	Unknown	Unknown	Guangdong	ORF5
8	HNLCL7-1804	OP302774	2018	Sow abortion rates of 10%	1000	Henan	ORF5
9	GDXNF94-1804	OP302758	2018	Unknown	Unknown	Guangdong	ORF5
10	TJWK169-1804	OP302773	2018	Unknown	Unknown	Tianjin	ORF5
11	GDXNF161-1806	OP302759	2018	Unknown	500	Guangdong	ORF5
12	LNDB50-1806	OP302770	2018	Unknown	Unknown	Liaoning	ORF5
13	HLJZD25-1810	OP302764	2018	Unknown	Unknown	Heilongjiang	ORF5
14	HNLCL53-1812	OP302772	2018	Sow abortion rates of 10%	300	Henan	ORF5
15	HNLCL75-1812	OP302767	2018	Unknown	Unknown	Henan	ORF5
16	HLJTZJ155-2001	OP302765	2020	Unknown	Unknown	Heilongjiang	ORF5
17	XJTZJ158-2001	OP302775	2020	Unknown	Unknown	Xinjiang	ORF5
18	HLJWK335-2005	OP302769	2020	Unknown	Unknown	Heilongjiang	ORF5
19	TZJ642	OP302763	2020	Piglet mortality rate of 2.6%	500	Henan	ORF5
20	SDHSW160-2201	OP302760	2022	Unknown	Unknown	Shandong	ORF5

**Table 2:** Comparison of pathogenicity of different PRRSV-1 strains.

Infected PRRSV strain	Subtype	Country	The days of inoculation (dpi)	Inoculated dose	Parameters of evaluation	Challenge group	Reference
ZD-1	Subtype 1	China	21	4 × 10^4^ TCID_50_	Clinical symptoms	Medium clinical symptoms	This study
					Days of fever	6 days (≥40.0°C)	
					Pathological and histopathological lesions	Obvious pathological changes	
					Viremia	Peaked at 7 dpi, longer than 21 days	

HLJB1	Subtype 1	China	14	1 × 10^5^ TCID_50_	Clinical symptoms	Severe clinical symptoms, 3/7 pigs died	[16]
					Days of fever	3 days (≥40°C)	
					Pathological and histopathological lesions	Obvious pathological changes	
					Viremia	Peaked at 7 dpi, longer than 21 days	

GZ11-G1	Subtype 1	China	21	2 × 10^5^ TCID_50_	Clinical symptoms	Mild clinical symptoms	[17]
					Days of fever	3 days (≥40.0°C)	
					Pathological and histopathological lesions	Mild pathological changes	
					Viremia	Peaked at 7 dpi, longer than 21 days	

PR40/2014	Subtype 1	Italy	35	1 × 10^5^ TCID_50_	Clinical symptoms	Severe clinical symptoms, 3/7 pigs died	[49]
					Days of fever	19 days (≥40°C)	
					Pathological and histopathological lesions	Severe pathological changes	
					Viremia	Peaked at 7 dpi, longer than 35 days	

WestSib13	Subtype 2	Russia	14	5 × 10^4.7^ TCID_50_	Clinical symptoms	Severe clinical symptoms, 5/5 pigs died	[52]
					Days of fever	No fever	
					Pathological and histopathological lesions	Severe pathological changes	
					Viremia	Peaked at 6 dpi, all pigs died seven days after infection	

Lena	Subtype 3	Belarusian	21	1 × 10^6^ TCID_50_	Clinical symptoms	Severe clinical symptoms, 4/10 pigs died	[45]
					Days of fever	25 days (≥40°C)	
					Pathological and histopathological lesions	Severe pathological changes	
					Viremia	Peaked at 14 dpi, longer than 35 days	

## Data Availability

The original contributions presented in the study are included in the article; further inquiries can be directed to the corresponding authors.

## References

[1] Brinton M. A., Gulyaeva A. A., Balasuriya U. B. R. (2021). ICTV virus taxonomy profile: Arteriviridae 2021. *Journal of General Virology*.

[2] Nathues H., Alarcon P., Rushton J. (2017). Cost of porcine reproductive and respiratory syndrome virus at individual farm level - an economic disease model. *Preventive Veterinary Medicine*.

[3] Collins J. E., Benfield D. A., Christianson W. T. (1992). Isolation of swine infertility and respiratory syndrome virus (isolate ATCC VR-2332) in North America and experimental reproduction of the disease in gnotobiotic pigs. *Journal of Veterinary Diagnostic Investigation*.

[4] Wensvoort G., de Kluyver E. P., Pol J. M. (1992). Lelystad virus, the cause of porcine epidemic abortion and respiratory syndrome: a review of mystery swine disease research at Lelystad. *Veterinary Microbiology*.

[5] Chen N., Xiao Y., Ye M. (2020). High genetic diversity of Chinese porcine reproductive and respiratory syndrome viruses from 2016 to 2019. *Research in Veterinary Science*.

[6] Guo Z., Chen X. X., Li R., Qiao S., Zhang G. (2018). The prevalent status and genetic diversity of porcine reproductive and respiratory syndrome virus in China: a molecular epidemiological perspective. *Virology Journal*.

[7] Li C., Xu H., Zhao J. (2022). Epidemiological investigation and genetic evolutionary analysis of PRRSV-1 on a pig farm in China. *Frontiers in Microbiology*.

[8] Liu J. K., Wei C. H., Yang X. Y. (2013). Genetic diversity and evolutionary characterization of Chinese porcine reproductive and respiratory syndrome viruses based on NSP2 and ORF5. *Archives of Virology*.

[9] Sun Q., Xu H., Li C. (2022). Emergence of a novel PRRSV-1 strain in mainland China: a recombinant strain derived from the two commercial modified live viruses Amervac and DV. *Frontiers in Veterinary Science*.

[10] Xu H., Song S., Zhao J. (2020). A potential endemic strain in China: NADC34-like porcine reproductive and respiratory syndrome virus. *Transbound Emerging Disease*.

[11] Zhang Q., Song Z., Yu Y., Huang J., Jiang P., Shan H. (2020). Genetic analysis of a porcine reproductive and respiratory syndrome virus 1 strain in China with new patterns of amino acid deletions in nsp2, GP3 and GP4. *Microbial Pathogenesis*.

[12] Balka G., Podgorska K., Brar M. S. (2018). Genetic diversity of PRRSV 1 in Central Eastern Europe in 1994-2014: origin and evolution of the virus in the region. *Scientific Reports*.

[13] Stadejek T., Oleksiewicz M. B., Potapchuk D., Podgorska K. (2006). Porcine reproductive and respiratory syndrome virus strains of exceptional diversity in eastern Europe support the definition of new genetic subtypes. *Journal of General Virology*.

[14] Stadejek T., Oleksiewicz M. B., Scherbakov A. V. (2008). Definition of subtypes in the European genotype of porcine reproductive and respiratory syndrome virus: nucleocapsid characteristics and geographical distribution in Europe. *Archives of Virology*.

[15] Stadejek T., Stankevicius A., Murtaugh M. P., Oleksiewicz M. B. (2013). Molecular evolution of PRRSV in Europe: current state of play. *Veterinary Microbiology*.

[16] Ming S., Yongying M., Bohua L. (2017). Pathogenic characterization of European genotype porcine reproductive and respiratory syndrome virus recently isolated in mainland China. *The Open Virology Journal*.

[17] Wang X., Yang X., Zhou R. (2016). Genomic characterization and pathogenicity of a strain of type 1 porcine reproductive and respiratory syndrome virus. *Virus Research*.

[18] Zhang H., Leng C., Feng L. (2015). A new subgenotype 2.1d isolates of classical swine fever virus in China, 2014. *Infection, Genetics and Evolution*.

[19] Zhang H., Zhang J., Zhang W. (2017). Complete genomic characterization of three European genotype porcine reproductive and respiratory syndrome viruses from China in 2016. *Zhong Guo Yu Fang Shou Yi Xue Bao/Chinese Journal of Preventive Veterinary Medicine*.

[20] Xu H., Li C., Li W. (2022). Novel characteristics of chinese nadc34-like prrsv during 2020-2021. *Transbound Emerging Disease*.

[21] Katoh K., Standley D. M. (2013). MAFFT multiple sequence alignment software version 7: improvements in performance and usability. *Molecular Biology and Evolution*.

[22] Xu H., Xiang L., Tang Y. D. (2022b). Genome-wide characterization of QYYZ-like PRRSV during 2018-2021. *Frontiers in Veterinary Science*.

[23] Kumar S., Stecher G., Tamura K. (2016). MEGA7: molecular evolutionary genetics analysis version 7.0 for bigger datasets. *Molecular Biology and Evolution*.

[24] Letunic I., Bork P. (2021). Interactive Tree of Life (iTOL) v5: an online tool for phylogenetic tree display and annotation. *Nucleic Acids Research*.

[25] Ramos N., Mirazo S., Castro G., Arbiza J. (2013). Molecular analysis of Porcine Circovirus Type 2 strains from Uruguay: evidence for natural occurring recombination. *Infection, Genetics and Evolution*.

[26] Kosakovsky Pond S. L., Posada D., Gravenor M. B., Woelk C. H., Frost S. D. W. (2006). Automated phylogenetic detection of recombination using a genetic algorithm. *Molecular Biology and Evolution*.

[27] Lole K. S., Bollinger R. C., Paranjape R. S. (1999). Full-length human immunodeficiency virus type 1 genomes from subtype C-infected seroconverters in India, with evidence of intersubtype recombination. *Journal of Virology*.

[28] de Abin M. F., Spronk G., Wagner M., Fitzsimmons M., Abrahante J. E., Murtaugh M. P. (2009). Comparative infection efficiency of Porcine reproductive and respiratory syndrome virus field isolates on MA104 cells and porcine alveolar macrophages. *Canadian Journal of Veterinary Research*.

[29] Song S., Xu H., Zhao J. (2020). Pathogenicity of NADC34-like PRRSV HLJDZD32-1901 isolated in China. *Veterinary Microbiology*.

[30] Zhang H., Leng C., Ding Y. (2019). Characterization of newly emerged NADC30-like strains of porcine reproductive and respiratory syndrome virus in China. *Archives of Virology*.

[31] Leng C. L., An T. Q., Chen J. Z. (2012). Highly pathogenic porcine reproductive and respiratory syndrome virus GP5 B antigenic region is not a neutralizing antigenic region. *Veterinary Microbiology*.

[32] Wang Q., Chen J., Peng J. (2014). Characterisation of novel linear antigen epitopes on North American-type porcine reproductive and respiratory syndrome virus M protein. *Archives of Virology*.

[33] Zhang H., Xiang L., Xu H. (2022). Lineage 1 porcine reproductive and respiratory syndrome virus attenuated live vaccine provides broad cross-protection against homologous and heterologous nadc30-like virus challenge in piglets. *Vaccines*.

[34] Chen N., Liu Q., Qiao M., Deng X., Chen X., Sun M. (2017). Whole genome characterization of a novel porcine reproductive and respiratory syndrome virus 1 isolate: genetic evidence for recombination between Amervac vaccine and circulating strains in mainland China. *Infection, Genetics and Evolution*.

[35] Ropp S. L., Wees C. E. M., Fang Y. (2004). Characterization of emerging European-like porcine reproductive and respiratory syndrome virus isolates in the United States. *Journal of Virology*.

[36] Chen N., Cao Z., Yu X. (2011). Emergence of novel European genotype porcine reproductive and respiratory syndrome virus in mainland China. *Journal of General Virology*.

[37] Liu J. K., Wei C. H., Dai A. L. (2017). Complete genomic characterization of two European-genotype porcine reproductive and respiratory syndrome virus isolates in Fujian province of China. *Archives of Virology*.

[38] Zhai S. L., Lin T., Zhou X. (2018). Phylogeographic analysis of porcine reproductive and respiratory syndrome virus 1 in Guangdong province, Southern China. *Archives of Virology*.

[39] Zhao J., Zhu L., Deng H. (2021). Genetic characterization of a novel porcine reproductive and respiratory syndrome virus type I strain from southwest China. *Archives of Virology*.

[40] Zhou Z., Liu Q., Hu D. (2015). Complete genomic characterization and genetic diversity of four European genotype porcine reproductive and respiratory syndrome virus isolates from China in 2011. *Virus Genes*.

[41] Kim S. H., Roh I. S., Choi E. J. (2010). A molecular analysis of European porcine reproductive and respiratory syndrome virus isolated in South Korea. *Veterinary Microbiology*.

[42] Vanhee M., Costers S., Van Breedam W., Geldhof M. F., Van Doorsselaere J., Nauwynck H. J. (2010). A variable region in GP4 of European-type porcine reproductive and respiratory syndrome virus induces neutralizing antibodies against homologous but not heterologous virus strains. *Viral Immunology*.

[43] Forsberg R., Oleksiewicz M. B., Krabbe Petersen A. M., Hein J., Botner A., Storgaard T. (2001). A molecular clock dates the common ancestor of European-type porcine reproductive and respiratory syndrome virus at more than 10 years before the emergence of disease. *Virology*.

[44] Wang A., Zhang J., Shen H. (2019). Genetic diversity of porcine reproductive and respiratory syndrome virus 1 in the United States of America from 2010 to 2018. *Veterinary Microbiology*.

[45] Karniychuk U. U., Geldhof M., Vanhee M., Van Doorsselaere J., Saveleva T. A., Nauwynck H. J. (2010). Pathogenesis and antigenic characterization of a new East European subtype 3 porcine reproductive and respiratory syndrome virus isolate. *BMC Veterinary Research*.

[46] Chae C. (2021). Commercial prrs modified-live virus vaccines. *Vaccines (Basel)*.

[47] Lin W. H., Kaewprom K., Wang S. Y. (2020). Outbreak of porcine reproductive and respiratory syndrome virus 1 in taiwan. *Viruses*.

[48] Lyoo K. S., Yeom M., Choi J. Y., Park J. H., Yoon S. W., Song D. (2015). Unusual severe cases of type 1 porcine reproductive and respiratory syndrome virus (PRRSV) infection in conventionally reared pigs in South Korea. *BMC Veterinary Research*.

[49] Canelli E., Catella A., Borghetti P. (2017). Phenotypic characterization of a highly pathogenic Italian porcine reproductive and respiratory syndrome virus (PRRSV) type 1 subtype 1 isolate in experimentally infected pigs. *Veterinary Microbiology*.

[50] Frydas I. S., Nauwynck H. J. (2016). Replication characteristics of eight virulent and two attenuated genotype 1 and 2 porcine reproductive and respiratory syndrome virus (PRRSV) strains in nasal mucosa explants. *Veterinary Microbiology*.

[51] Martin-Valls G. E., Cortey M., Allepuz A., Illas F., Tello M., Mateu E. (2022). Description of a new clade within subtype 1 of Betaarterivirus suid 1 causing severe outbreaks in Spain. *Microbiol Resour Announc*.

[52] Yuzhakov A. G., Raev S. A., Skrylev A. N. (2017). Genetic and pathogenic characterization of a Russian subtype 2 PRRSV-1 isolate. *Veterinary Microbiology*.

[53] Morgan S. B., Graham S. P., Salguero F. J. (2013). Increased pathogenicity of European porcine reproductive and respiratory syndrome virus is associated with enhanced adaptive responses and viral clearance. *Veterinary Microbiology*.

[54] Zhou L., Kang R., Yu J. (2018). Genetic characterization and pathogenicity of a novel recombined porcine reproductive and respiratory syndrome virus 2 among nadc30-like, jxa1-like, and mlv-like strains. *Viruses*.

[55] Zhao Y., Li Z., Chen R., Luo C. (1998). Molecular cloning and identification of the ORF7 gene of Chinese isolate B13 of porcine reproductive and respiratory syndrome virus (PRRSV). *Chinese Journal of Veterinary Medicine*.

